# *In Vitro* and *in Vivo* Anti-Hyperglycemic Effects of Omija (*Schizandra chinensis*) Fruit

**DOI:** 10.3390/ijms12021359

**Published:** 2011-02-23

**Authors:** Sung-Hoon Jo, Kyoung-Soo Ha, Kyoung-Sik Moon, Ok-Hwan Lee, Hae-Dong Jang, Young-In Kwon

**Affiliations:** 1 Department of Food and Nutrition, Hannam University, Daejeon 305-811, Korea; E-Mails: dutnskfk@hanmail.net (S.-H. J.); kengkoo@nate.com (K.-S. H.); Haedong@hnu.kr (H.-D. J.); 2 Division of Non-Clinical Studies, Korea Institute of Toxicology, Daejeon 305-343, Korea; E-Mail: ksmoon@kitox.re.kr; 3 Department of Food Science and Biotechnology, Kangwon National University, Chuncheon, 200-701, Korea; E-Mail: loh99@hanmail.net

**Keywords:** α-glucosidase, antihyperglycemia, blood glucose, oxygen radical absorbance capacity, Schizandra chinensis

## Abstract

The entrocytes of the small intestine can only absorb monosaccharides such as glucose and fructose from our diet. The intestinal absorption of dietary carbohydrates such as maltose and sucrose is carried out by a group of α-glucosidases. Inhibition of these enzymes can significantly decrease the postprandial increase of blood glucose level after a mixed carbohydrate diet. Therefore, the inhibitory activity of Omija (*Schizandra chinensis*) extract against rat intestinal α-glucosidase and porcine pancreatic α-amylase were investigated *in vitro* and *in vivo*. The *in vitro* inhibitory activities of water extract of Omija pulp/skin (OPE) on α-glucosidase and α-amylase were potent when compared to Omija seeds extract (OSE). The postprandial blood glucose lowering effect of Omija extracts was compared to a known type 2 diabetes drug (Acarbose), a strong α-glucosidase inhibitor in the Sprague-Dawley (SD) rat model. In rats fed on sucrose, OPE significantly reduced the blood glucose increase after sucrose loading. Furthermore, the oxygen radical absorbance capacity (ORAC) of OSE and OPE was evaluated. OPE had higher peroxyl radical absorbing activity than OSE. These results suggest that Omija, which has high ORAC value with α-glucosidase inhibitory activity and blood glucose lowering effect, could be physiologically useful for treatment of diabetes, although clinical trials are needed.

## 1. Introduction

Non-insulin dependent diabetes mellitus (NIDDM, type 2 diabetes) is a common disorder of glucose and fat metabolism that affects 171 million people worldwide, generating immense health care costs [1]. Hyperglycemia, a typical symptom in NIDDM patients, is a condition characterized by a rapid rise in blood glucose levels and is due to hydrolysis of starch by pancreatic α-amylase and absorption of glucose in the small intestine by α-glucosidases [2]. One of the therapeutic approaches for decreasing postprandial hyperglycemia is to retard digestion of glucose by the inhibition of carbohydrate hydrolyzing enzymes, α-amylase and α-glucosidase, in the digestive tract [2]. Therefore, inhibition of these carbohydrate-hydrolyzing enzymes can significantly decrease the postprandial hyperglycemia after a mixed carbohydrate diet and can be a key strategy in the control of diabetes mellitus [3].

Postprandial hyperglycemia has been linked to the onset of diabetic complications in NIDDM patients and triggers the generation of free radicals and oxidation-related damage in the retina, renal glomerulus and peripheral nerves [4,5]. Studies have shown that the glucose-induced increased levels of mitochondrial reactive oxygen species (ROS) produced by the mitochondrial electron transport chain seems to be the causal link between elevated levels of glucose and the pathways responsible for hyperglycemia-induced vascular complications [4,6]. Therefore, it is important to control both cellular redox status and blood glucose level for managing these diabetic complications.

*Schizandra chinensis* is known as Omija in Korea, which literally means berry with five different flavors [7]. Its seeds and fruits have been used as a traditional medicinal plant in Asia. Recent research has reported that phenolic phytochemicals from Omija (*S. chinensis*) have high antioxidant activity and anti-inflammatory effects in *in vitro* and *in viv*o models [7,8]. Furthermore it has been reported that Omija fruit and leaves are rich in anthocyans such as anthocyanins, which have perceived benefits to human health [7,9]. Therefore, in this study the seeds and pulp were separated from whole Omija fruit and used to evaluate antioxidant and anti-hyperglycemic activities.

Epidemiological studies have also shown that the intake of certain types of anthocyanins-rich cherries is inversely associated with the risk of incident oxidative stress-linked diseases [10]. Additionally, it has been reported that dietary anthocyanin-rich bilberry extract ameliorates insulin sensitivity in diabetic mice [11]. However, there is little information on the antihyperglycemic properties of the Omija extracts.

Therefore, the aim of this study is to investigate the mode of action and effect of Omija extract on the type 2 diabetes management related inhibition of postprandial hyperglycemia in *in vitro* and *in vivo* animal model. Clear knowledge of the activity and mode of action of Omija extract will contribute towards better understanding of the real effect of various Omija products towards type 2 diabetes management. To determine the above, in this study, we (i) prepared Omija extracts (OSE; Omija seeds extract, OPE; Omija pulp/skin extract) by water extraction; (ii) investigated the inhibitory activity of OSE and OPE against α-amylase and α-glucosidase (anti-hyperglycemia potential); (iii) measured antioxidant potential using oxygen radical scavenging capacity (ORAC) assay, and (iv) evaluated the postprandial blood glucose lowering effect of OSE and OPE after sucrose loading in a Sprague-Dawley (SD) rat model.

## 2. Results and Discussion

### 2.1. a-Amylase Inhibition

The α-amylase inhibitors, which interfere with enzymatic action in the small intestine, could slow the liberation of maltose from starch, resulting in delaying maltose conversion to glucose and decreasing postprandial plasma glucose levels [9]. Recent research with phenolic enriched herbal extracts reported an association between α-amylase and α-glucosidase inhibitory activity [12]. All the herb extracts showed a comparable inhibition of α-glucosidase but did not have any inhibitory activity against porcine pancreatic α-amylase. Therefore, we evaluated the inhibitory activity of water extracts of Omija fruit (OSE; Omija seeds extract, OPE; Omija pulp/skin extract) against α-amylase from porcine pancreas in this study.

**Figure 1.**
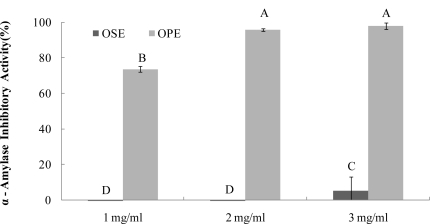
Dose dependent changes in porcine pancreatic α-amylase inhibitory activity (% inhibition) of Omija extract (water extract of Omija seeds: OSE, water extract of Omija pulp/skin: OPE). The results represent the mean ± SD. of values obtained from three measurements. Different corresponding letters indicate significant differences at *p* < 0.05 by Duncan’s test.

As seen in Figure 1, OPE showed potent α-amylase inhibitory activity (74%) followed by OSE (2%), (Figure 1) at the same concentration (1 mg/mL). OPE showed a comparable inhibition (IC_50_; <1.0 mg/mL) against α-amylase but OSE did not have significant α-amylase inhibitory activity.

### 2.2. a-Glucosidase Inhibition

**Figure 2.**
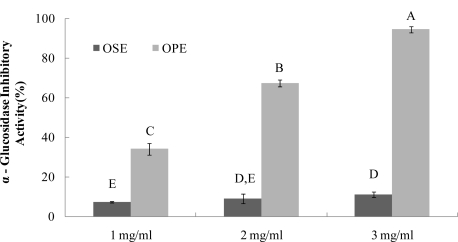
Dose dependent changes in rat intestinal α-glucosidase inhibitory activity (% inhibition) of Omija extracts (water extract of Omija seeds: OSE, water extract of Omija pulp/skin: OPE). The results represent the mean ± SD. of values obtained from three measurements. Different corresponding letters indicate significant differences at *p* < 0.05 by Duncan’s test.

The α-glucosidase inhibitors, which interfere with enzymatic action in the brush-border of the small intestine, could inhibit the liberation of d-glucose from oligosaccharides and disaccharides, resulting in delaying glucose absorption and decreasing postprandial plasma glucose levels [9]. Previous research with onion extracts reported that methyl alcohol extracts of onion had high microbial α-glucosidase (from Baker’s yeast) inhibitory activity [13]. It have been reported that most yeast α-glucosidase inhibitors did not show inhibitory activity against mammalian α-glucosidase due to the difference of molecular recognition in the binding site of the enzymes [14], Therefore, in order to have better health relevance, mammalian α-glucosidase (from rat intestine) was used to estimate the inhibitory activities of OSE and OPE in this study. The OPE had potent α-glucosidase inhibitory activity with an IC_50_ value of 1.49 mg/mL (Figure 2), indicating a potential role as an antidiabetic natural source. As a result, OSE showed weak α-glucosidase inhibitory activities with IC_50_ values of >3.00 mg/mL, whereas no α-amylase inhibition was observed (Figures 1 and 2).

Our previous results showed that individual phenolic compounds had α-glucosidase inhibitory activity [5]. This previous result indicates that individual phenolic compounds play a role in the inhibition of α-glucosidase inhibitory activity. Further, another research group reported that one of the major phenolic phytochemicals in Omija anthocyanin acts as a competitive α-glucosidase inhibitor [15,16]. These results were in good agreement with the high α-glucosidase and α-amylase inhibitory activities of OPE compared to OSE, since the content of anthocyanin in Omija pulp is higher than that of Omija seed in general.

Based on these results, data trends for α-amylase and α-glucosidase inhibitory activities in OPE have important implications for the development and new design of Omija-based functional foods. This has clear potential for inhibiting carbohydrate-hydrolyzing enzymes using the specific enrichment of ingredients in OPE instead of OSE.

### 2.3. Antioxidant Activity by ORAC System

**Figure 3.**
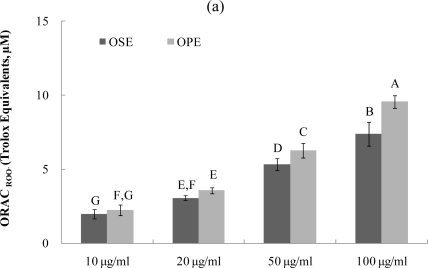

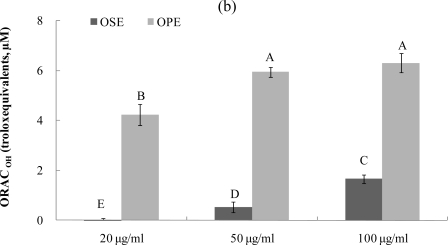
Dose dependent changes in peroxyl and hydroxyl radical scavenging activities (Trolox equivalent, μM) of Omija extracts in *in vitro* system (water extract of Omija seeds: OSE, water extract of Omija pulp/skin: OPE). (**a**) Peroxyl radical scavenging activity of extracts of Omija; (**b**) hydroxyl radical scavenging activity of extracts of Omija. The oxygen radical absorbance capacity (ORAC) value is calculated by dividing the area under the sample curve by the area under the Trolox curve, with both areas being corrected by subtracting the area under the blank curve. One ORAC unit is assigned as the net area of protection provided by Trolox at a final concentration of 1 μM. The area under the curve for the sample is compared to the area under the curve for Trolox, and the anti-oxidative value is expressed in micromoles of Trolox equivalent per liter. The results represent the mean ±SD. of values obtained from three measurements. Different corresponding letters indicate significant differences at *p* < 0.05 by Duncan’s test.

The ORAC assay developed by Cao *et al*. [17–19] has been used successfully to determine the reaction capacity with peroxyl radical, one harmful and reactive oxygen species in biological systems. Antioxidant activity of Omija extracts was investigated for their peroxyl radical-scavenging capacity using the ORAC assay system, where AAPH was used as a generator of peroxyl radicals. Figure 3 demonstrates that the scavenging activity of Omija extract on peroxyl radicals generated from AAPH was found to be dose-dependent between 10 μg/mL and 100 μg/mL. The bars in Figure 3a represent the ORAC_ROO_ activity of 1 μM of the tested sample equivalent to 1 μM Trolox, a water-soluble α-tocopherol analogue. The ORAC values for the sample extracts ranged from 2.1 μM of Trolox equivalents (TE) to 9.5 μM of TE. The Omija extract (100 μg/mL) with high ORAC values were OPE (9.5 TE) and OSE (7.4 TE), respectively. The hydroxyl radical absorbing activity (ORAC_HO·_) of OPE and OSE was also measured using ORAC assay in which Cu^2+^ and H_2_O_2_ were used as hydroxyl radical generator. Compared to OSE, OPE showed potent ORAC_HO·_ value (Figure 3b; 6.5 TE) at 100 μg/mL concentration.

These data clearly demonstrate that the presence of phenolic phytochemicals in Omija may play an important role in antioxidant activity such as radical scavenging and (or) transition metal chelating, and result in a reduction of antioxidant activity, which is in agreement with previous study by Kim *et al.* [7]. Any dietary management of hyperglycemia linked to type 2 diabetes and related complications from oxidative dysfunction can benefit from specific enzyme inhibitory activity combined with antioxidant activity in the same whole food extracts. Insights from this study indicate that OPE have α-glucosidase inhibitory activity and high peroxyl radical scavenging-linked antioxidant activity and therefore have the potential to contribute to the reduction of hyperglycemia-induced microvascular complications.

### 2.4. *In Vivo* Blood Glucose Lowering Effect of Omija Extracts

OPE showed significant inhibition against α-glucosidases, which are membrane-bound enzymes at the epithelia of the small intestine and key enzymes of carbohydrate digestion [2]. Inhibition of these enzyme leads to a delayed and reduced rise in postprandial blood glucose levels.

**Figure 4.**
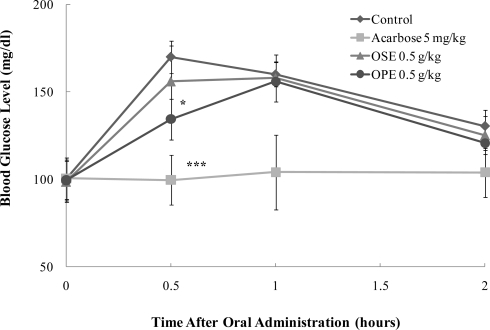
Effect of Omija extracts on sucrose loading test. After fasting for 24 hours, 6-week-old, male SD rats were orally administered with sucrose solution (2.0 g/kg) with or without samples (water extract of Omija seeds: OSE, water extract of Omija pulp/skin: OPE, positive control: Acarbose). Each point represents mean ± SD. (n = 5). *p < 0.05, **p < 0.01, and ***p < 0.001 compared to different samples at the same concentration by unpaired Student’s *t*-test.

To prove *in vitro* α-glucosidase inhibitory activity of OPE, the *in vivo* blood glucose reducing effect of OPE and OSE was evaluated with SD rats and the results are illustrated in Figure 4.

In SD rats, OPE exerted a statistically significant decrease (*p* < 0.05) of the blood glucose at half an hour after sucrose loading. OPE significantly reduced (*p* < 0.05) the postprandial hyperglycemia caused by sucrose loading to an extent less than that observed in the acarbose administered group (*p* < 0.001) (Figure 4).

The pharmacokinetic parameters of SD rats administered with OSE, OPE, and acarbose are shown in Table 1. The OPE treatment at 0.5 g/kg body weight significantly decreased area under the blood glucose-time curve (AUC) (*p* < 0.05) and C_max_ (*p* < 0.05) blood glucose in rats that ingested sucrose compared to control. On the other hand, T*_max_* significantly (*p* < 0.01) increased in rats treated with OPE compared to control when sucrose was orally administered to them.

These results may demonstrate the positive effects of OPE against hyperglycemia resulting from high sucrose ingestion. It is suggested that OPE with high blood glucose lowering effect may be used for the development of pharmaceutical food to control the blood glucose level of diabetic patients by inhibiting α-glucosidase and α-amylase in the intestinal tract.

Although, in this study, we provided evidence for OPE as a α-glucosidase inhibitor and its properties to decrease blood glucose at a dose of 0.5 g/kg-body weight, the exact potency and efficacy of OPE on postprandial hyperglycemia in a rat model is not yet clear, and the potential anti-hyperglycemic component in OPE remains to be investigated. Therefore, to provide the precise pharmacokinetics based on a single compound, further pharmacological and biological studies are needed.

## 3. Experimental Section

### 3.1. Materials

Omija (*Schizandra chinensis* Bail.) was purchased from Geumsan Omija farm Co. (Geumsan, Chungnam, Korea). The dried fruits of *S. chinensis* were identified by one of authors (Ok-Hwan Lee). A voucher specimen (BFC O10011) was deposited at the Bioactive Food Components Lab. (BFCL) of the College of Life Science and Nano Technology, Hannam University. Porcine pancreatic α-amylase (EC 3.2.1.1) and rat intestinal acetone powders of α-glucosidase (EC 3.2.1.20) were also purchased from Sigma-Aldrich Co. (St. Louis, MO, USA). Unless noted, all chemicals were purchased from Sigma-Aldrich Co. (St. Louis, MO, USA).

### 3.2. Preparation of Extracts

After peeling of Omija (*Schizandra chinensis* Bail.) with a knife, 100 g of seed and skin-pulp were mashed and stirred respectively in 1000 mL of distilled water at 100 °C for 30 min. The seed extract (OSE) and skin-pulp extract (OPE) were then filtered through a Whatman # 2 filter, centrifuged at 7000 × g for 1 h, vacuum-evaporated at 45 °C, freeze-dried and kept at −70 °C until analysis.

### 3.3. a-Amylase Inhibition Assay

To evaluate the potency of Omija (*Schizandra chinensis* Bail.) extracts the dose dependency of Omija seed extract (OSE) and Omija skin/pulp extract (OPE) on α-amylase was measured using different concentrations (between 1.0 and 3.0 mg/mL). Porcine pancreatic α-amylase inhibition referred to the method of Kwon *et al.* [20]. Sample solution (200 μL) and 0.02 M sodium phosphate buffer (pH 6.9 with 0.006 M sodium chloride, 500 μL) containing α-amylase solution (0.5 mg/mL, 5.0 MU/mL) were incubated at 25 °C for 10 min. After pre-incubation, 500 μL of a 1% starch solution in 0.02 M sodium phosphate buffer was added. The reaction mixture was then incubated at 25 °C for 10 min. The reaction was stopped with 1.0 mL of dinitrosalicylic acid (DNS). The reaction mixture was then incubated in a boiling water bath for 5 min and cooled to room temperature. The reaction mixture was then diluted after adding distilled water, and absorbance was measured at 540 nm with ELISA micro-plate reader (SUNRISE; Tecan Trading AG, Saltzburg, Austria).

$$\text{Inhibition}\;(\%)=\left(\left[\frac{\Delta A_{540}^{\text{Control}}-\Delta A_{540}^{\text{Extract}}}{\left[\Delta A_{540}^{\text{Control}}\right]}\right]\right)\times 100$$

### 3.4. a-Glucosidase Inhibition Assay

To evaluate the potency of Omija (*Schizandra chinensis* Bail.) extracts, the dose dependency of Omija seed extract (OSE) and Omija skin/pulp extract (OPE) on rat intestinal α-glucosidase was measured using different concentrations (between 1.0 and 3.0 mg/mL). Rat intestinal α-glucosidase assay referred to the method of Kwon *et al*. [21] with slight modification. A total of 1 g of rat-intestinal acetone powder was suspended in 3 mL of 0.9% saline, and the suspension was sonicated 12 times for 30 sec at 4 °C. After centrifugation (10,000 × g, 30 min, 4 °C), the resulting supernatant was used for the assay. Sample solution (50 μL) and 0.1 M phosphate buffer (pH 6.9, 100 μL) containing glucosidase solution (1.0 U/mL) was incubated at 25 °C for 10 min. After pre-incubation, 5 mM *p*-nitrophenyl-α-d-glucopyranoside solution (50 μL) in 0.1 M phosphate buffer (pH 6.9) was added to each well at timed intervals. The reaction mixtures were incubated at 25 °C for 5 min. Before and after incubation, absorbance was read at 405 nm and compared to a control which had 50 μL of buffer solution in place of the extract by micro-plate reader (SUNRISE; Tecan Trading AG, Saltzburg, Austria). The α-glucosidase inhibitory activity was expressed as inhibition % and was calculated as follows:

$$\text{Inhibition}\;(\%)=\left(\left[\frac{\Delta A_{405}^{\text{Control}}-\Delta A_{405}^{\text{Extract}}}{\left[\Delta A_{405}^{\text{Control}}\right]}\right]\right)\times 100$$

### 3.5. Oxygen Radical Absorbance Capacity (ORAC) Assay

Antioxidant activities of Omija (*Schizandra chinensis* Bail.) extracts in different concentrations (between 10 and 100 μg/mL) were investigated for their peroxyl and hydroxyl radical-scavenging capacities using ORAC assay system. The ORAC assay was carried out using a Tecan GENios multi-functional plate reader (GENios; Tecan Trading AG, Salzburg, Austria) with fluorescent filters (excitation wavelength: 485 nm, emission filter: 535 nm). In the final assay mixture, fluorescein (40 nM) was used as a target of free radical attack with either 2,2′-azobis (2-amidinopropane) dihydrochloride (AAPH, 20 mM) as a peroxyl radical generator in peroxyl radical-scavenging capacity (ORAC_ROO·_) assay [22] or with H_2_O_2_-CuSO_4_ (H_2_O_2_, 0.75%; CuSO_4_, 5 μM) as a hydroxyl radical generator in hydroxyl radical-scavenging capacity (ORAC_HO·_) assay [15]. Trolox (1 μM) was used as a control standard and prepared fresh on a daily basis. The analyzer was programmed to record the fluorescence of fluorescein every 2 min after AAPH or H_2_O_2_-CuSO_4_ was added. All fluorescence measurements were expressed relative to the initial reading. Final results were calculated based on the difference in the area under the fluorescence decay curve between the blank and each sample. All data were expressed as micromoles of Trolox equivalents (TE). One ORAC unit is equivalent to the net protection area provided by 1 μM of Trolox.

### 3.6. Sugar Loading Test

Effect on hyperglycemia induced by carbohydrate loads in Sprague-Dawley (SD) rats was determined by the inhibitory action of OSE, OPE and Acarbose on postprandial hyperglycemia. Five week-old male SD rats were purchased from Joongang Experimental Animal Co. (Seoul, Korea) and fed a solid diet (Samyang Diet Co., Seoul, Korea) for one week. The rats were housed in a ventilated room at 25 ± 2 °C with 50 ± 7% relative humidity, and under an alternating 12 hour light/dark cycle. After 6 groups of 5 male SD rats (180∼200 g) were fasted for 24 h, 2.0 g/kg of sucrose were orally administrated concurrently with 0∼500 mg/kg inhibitors (OSE, OPE or Acarbose). The blood samples were then taken from the tail after administration and blood glucose levels were measured at 0, 0.5, 1, and 2 hours. The glucose level in blood was determined by glucose oxidase method and compared with that of the control group, which had not taken the inhibitors. The parameters for blood glucose levels were calculated using WinNonLin program (Version 5.2.1, Pharsight Corporation, Cary, NC, USA). Maximum observed peak blood glucose level (C_max_) and the time at which it is observed (T_max_) were determined based on the observed data. Area under the blood glucose-time curve up to the last sampled time-point (AUC_last_) was estimated by the trapezoidal rule.

### 3.7. Statistical Analysis

All data are presented as mean ±SD. Statistical analyses were carried out using the statistical package SPSS (Statistical Package for Social Science, SPSS Inc., Chicago, IL, USA) program and significance of each group was verified with the analysis of One-way ANOVA followed by the Duncan’s test of *p* < 0.05.

## 4. Conclusions

A sudden increase in blood glucose levels, causing hyperglycemia in NIDDM is due to hydrolysis of starch by pancreatic α-amylase and absorption of glucose in the small intestine by α-glucosidase. Furthermore, hyperglycemia-induced microvascular complications are likely from oxidative dysfunction from mitochondrial reactive oxygen species (ROS). Therefore, it is important to control both cellular redox status and blood glucose level for managing these diabetic complications.

The water extract of Omija skin/pulp (OPE) has high α-amylase and α-glucosidase inhibitory activity and potent peroxyl radical scavenging-linked antioxidant activity. Sucrose loading test showed that OPE may reduce postprandial increases of blood glucose level by acting as an intestinal α-glucosidase inhibitor. The above benefits (anti-hyperglycemia and antioxidant activity) of OPE taken together could support the evidence that diets rich in fruits and vegetables are associated with lower incidences of oxidation-linked diseases such as diabetes [23–25].

These *in vitro* and *in vivo* studies therefore could provide the biochemical rationale for the benefit of Omija-based dietary supplement and the basis for further clinical study.

## Figures and Tables

**Table 1.** Pharmacokinetic parameters of SD control rats or after administration of OSE, OPE, and acarbose after sucrose ingestion.

		**PK parameters**		
		**AUC_last_ (mg/dL·h)**	**C_max_ (mg/dL)**	**T_max_ (h)**
Sucrose	Control	295.3 ± 7.2	171.2 ± 7.2	0.6 ± 0.2
	Acarbose (5 mg/kg)	205.1 ± 29.4***	111.0 ± 15.8***	1.2 ± 0.8***
	OSE (0.5 g/kg)	291.8 ± 22.0	173.4 ± 13.5	0.8 ± 0.3
	OPE (0.5 g/kg)	276.6 ± 16.3*	157.2 ± 10.5*	1.0 ± 0.0**
